# The cytosolic tail of the tumor marker protein Trop2 - a structural switch triggered by phosphorylation

**DOI:** 10.1038/srep10324

**Published:** 2015-05-18

**Authors:** Miha Pavšič, Gregor Ilc, Tilen Vidmar, Janez Plavec, Brigita Lenarčič

**Affiliations:** 1 Department of Chemistry and Biochemistry, Faculty of Chemistry and Chemical Technology, University of Ljubljana, Večna pot 113, SI-1000 Ljubljana, Slovenia; 2 Slovenian NMR Centre, National Institute of Chemistry, Hajdrihova 19, SI-1000 Ljubljana, Slovenia; 3 EN-FIST Centre of Excellence, Dunajska 156, SI-1000 Ljubljana, Slovenia; 4 J. Stefan Institute, Department of Biochemistry, Molecular and Structural Biology, Jamova 39, SI-1000 Ljubljana, Slovenia

## Abstract

Trop2 is a transmembrane signaling glycoprotein upregulated in stem and carcinoma cells. Proliferation-enhancing signaling involves regulated intramembrane proteolytic release of a short cytoplasmic fragment, which is later engaged in a cytosolic signaling complex. We propose that Trop2 function is modulated by phosphorylation of a specific serine residue within this cytosolic region (Ser303), and by proximity effects exerted on the cytosolic tail by Trop2 dimerization. Structural characterization of both the transmembrane (Trop2TM) and cytosolic regions (Trop2IC) support this hypothesis, and shows that the central region of Trop2IC forms an α-helix. Comparison of NMR structures of non-phosphorylated and phosphorylated forms suggest that phosphorylation of Trop2IC triggers salt bridge reshuffling, resulting in significant conformational changes including ordering of the C-terminal tail. In addition, we demonstrate that the cytosolic regions of two Trop2 subunits can be brought into close proximity via transmembrane part dimerization. Finally, we show that Ser303-phosphorylation significantly affects the structure and accessibility of functionally important regions of the cytosolic tail. These observed structural features of Trop2 at the membrane-cytosol interface could be important for regulation of Trop2 signaling activity.

The tumor-associated calcium signal transducer 2 (Trop2) is a transmembrane type-1 glycoprotein expressed in multistratified epithelia and several stem/progenitor and carcinoma cells^1^,^2^,^3^,^4^. Trop2 transmits extracellular signals via its extracellular and transmembrane (Trop2TM) domains to a short (26 aa) cytosolic tail (Trop2IC)^5^. The extracellular domain (Trop2EC, 248 aa), which contains a small disulfide-rich unique domain, a tyroglobulin type-1 (TY) domain, and a cysteine-poor domain, is the largest topological unit. Published data show that Trop2EC forms a stable dimer^6^. Recently, the crystal structure of a dimer of the extracellular domain of its paralogue, human EpCAM, has been described^7^. The two triangularly-shaped subunits are held together by extensive interactions between the TY domain and the cysteine-poor domain, and the dimerization interface is believed to extend to the transmembrane region. Based on the high overall sequence identity between the two paralogues (~50%), we believe that Trop2 could form an analogous dimer (Fig. 1). While cell-cell adhesive interactions have to date been reported for EpCAM^8^, the two molecules have a signaling function that is generally similar: release of both extracellular and cytosolic parts, involvement of the cytosolic part in a signaling complex, and activation/enhanced transcription of cell cycle promoting genes^5^,^9^. In addition, both proteins were found to be associated with claudins and implicated in maintaining tight junction integrity^10^,^11^,^12^. For EpCAM, another interaction involving its transmembrane region and claudin-7 was reported: association with tetraspanin-enriched lipid microdomains^13^,^14^,^15^. Adding to the list of interactions, Trop2 also interferes with IGF-1R signaling by binding IGF1 via a thyroglobulin type-1 domain within Trop2EC^16^; and disrupts integrin α5β1-fibronectin contacts via interaction with the integrin β1 subunit^17^. Furthermore, due to elevated expression levels in carcinoma cells vs. normal tissue, Trop2 has clinical potential both for cancer prognosis^18^,^19^ and immunotherapy^20^,^21^.

Signaling-associated fragmentation of Trop2 involves the action of TNF-α-converting enzyme (TACE) and γ-secretase. Released Trop2IC then enters a β-catenin-dependent signaling cascade involving nuclear translocation of the Trop2IC/β-catenin complex. Nuclear colocalization of Trop2 and β-catenin is restricted to malignant tissue and is associated with enhanced activity of cell cycle promoting genes^5^. The importance of Trop2IC in signaling is also supported by a Trop2IC deletion mutant which abolishes signaling through ERK, NF-κB and cyclin D1^22^. Additionally, the activity of Trop2IC can be regulated via phosphorylation on Ser303 (ref. 23) and binding of phosphatidylinositol 4,5-bisphosphate (PIP_2_) to a region encompassing Lys302-Lys308 (ref. 24). Studies using PKC from rat brain suggest that PKC α-, β- and/or γ-subtypes could be involved in Trop2IC phosphorylation, however the exact PKC subtype and extent of Trop2IC phosphorylation *in vivo* remain to be determined^23^. Although Ser303 is highly conserved, there is no equivalent serine residue in EpCAM. Interestingly, mutating Ser303 inhibits Trop2 tumor growth stimulatory activity^25^. Recently, another PKC-involving function of Trop2 and EpCAM has been described where cytosolic parts of both molecules were shown to inhibit PKCδ^26^. Modeling suggests that the residue facing the catalytic site is Lys305, which results in a pseudosubstrate-like interaction between the cytosolic part of EpCAM/Trop2 and the active site of PKCδ. Adding to the complexity of these signaling events, oligomerization of Trop2 via its ectodomain^6^ (in a similar manner to EpCAM^7^) could influence initial signaling events by bringing the two transmembrane and cytosolic regions in close proximity.

Both Ser303 phosphorylation and the spatial proximity exerted by transmembrane part dimerization could play important roles in modulation of Trop2IC function and possibly the function of Trop2 as a whole. To gain insight into these possibilities at an atomic level, we performed detailed structural analysis of Trop2IC and the transmembrane domain. First, we studied the secondary structures of both forms using a secondary-structure stabilizing co-solvent, 2,2,2-trifluoroethanol (TFE). There are many examples where TFE-induced peptide structure is physiologically relevant in terms of protein-protein interactions and function^27^,^28^,^29^,^30^,^31^. Next, we determined the structures of both non-phosphorylated and phosphorylated forms of Trop2IC (Trop2IC and Trop2ICP, respectively) in buffer/TFE mixture by NMR spectroscopy. Finally, we used molecular dynamics simulations of Trop2TM embedded in a lipid bilayer to assess their dimerization potential. Our results demonstrate that phosphorylation induces structural changes in Trop2IC via a charge re-distribution mechanism and that the Trop2 dimerization interface extends to the transmembrane part.

## Results and Discussion

**Figure 1:**
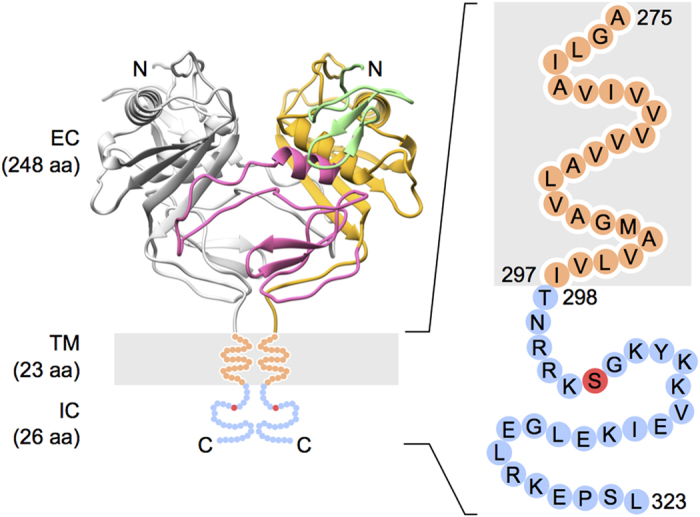
Domain organization of Trop2. Using a homology model of a Trop2 dimer, the extracellular domain of one subunit of Trop2 (left) is colored according to domain boundaries (N-terminal cysteine-rich domain in green, TY domain in pink, and C-terminal cysteine-poor domain in yellow), while the other Trop2 subunit is colored gray. The transmembrane (TM) and the intracellular tail (IC) are shown in orange and blue, respectively, and the conserved phosphorylation site (Ser303) within the IC is shown in red.

Detailed studies of complex transmembrane proteins such as Trop2 (Fig. 1) are associated with potential problems regarding lipid/detergent embedded protein regions and significant conformational flexibility. Because of this, we studied the transmembrane (Trop2TM) and cytosolic regions (Trop2IC) of Trop2 separately. For Trop2IC and Trop2ICP, we used a synthetic peptide corresponding to the cytosolic part of Trop2, while for Trop2TM we used an *in silico* approach.

### Addition of TFE enables detection of α-helical structure in the Trop2 cytoplasmic region by CD

**Figure 2:**
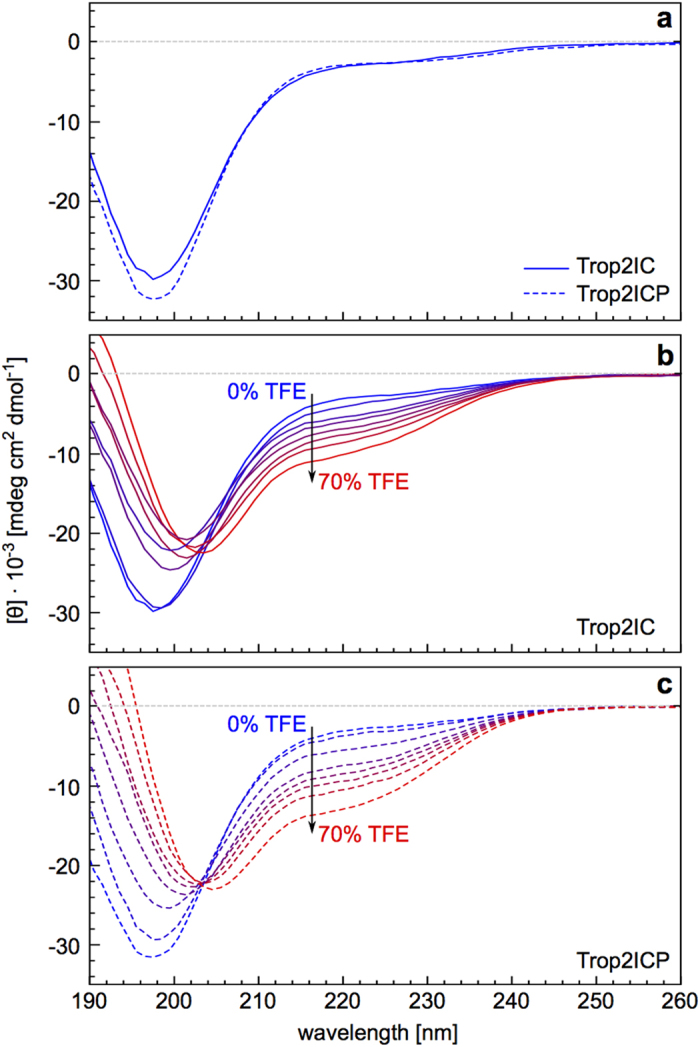
Effect of TFE on far-UV CD spectra of Trop2IC and Trop2ICP. TFE induces helical structure in both peptides (**b, c**) as compared to aqueous solution (**a**). Additions of TFE were performed stepwise (10% each).

To obtain a general view of the structural differences between Trop2IC and Trop2ICP, we first analyzed both peptides by circular dichroism (CD), in phosphate buffer (pH 7.4) supplemented with varying amounts of TFE. CD spectra of both Trop2IC and Trop2ICP in phosphate buffer without TFE exhibit a significant minimum at 198 nm, indicating that both peptides lack significant secondary structure (Fig. 2a). Addition of 2,2,2-trifluoroethanol (TFE, a well-known secondary structure stabilizing agent^32^) to a final concentration of 70% gradually increased the level of detectable helical structure in both peptides, as indicated by a shift of the minimum to 208 nm coupled with the appearance of an additional minimum at 222 nm (Fig. 2b,c), which is characteristic of an α-helix. Total α-helical content in Trop2ICP is somewhat larger (39%) than in Trop2IC (29%), as calculated from CD spectra recorded in 70% TFE. This is in general agreement with secondary structure prediction using PSIPRED^33^,^34^ where a nine residue long α-helix is predicted (Glu310-Arg318) with medium confidence (on average 43% per residue) giving a net 35% α-helical content. Measurements at even higher TFE concentrations were not possible due to peptide insolubility.

Results from CD measurements in phosphate buffer alone could indicate that the helical structure observed in TFE is only an artifact, and therefore does not reflect the intrinsic propensity of the peptide to form an α-helix in aqueous solution without TFE. However, the absence of detectable secondary structure could also be a consequence of instability, in agreement with the relatively low confidence of secondary structure prediction for this region. For example, cytoplasmic domains of connexins have α-helical structure as part of the complete membrane-anchored molecule, or when membrane-tethered, but isolated cytoplasmic domains exhibit detectable helical structure only in aqueous solution containing TFE^35^,^36^,^37^. Similarly, the helix-stabilizing effect of TFE in hen lysozyme is observed only in those regions of the polypeptide chain that intrinsically tend to form helical secondary structure elements^38^.

### NMR spectra indicate α-helical structure for Trop2IC in the absence of TFE

**Figure 3:**
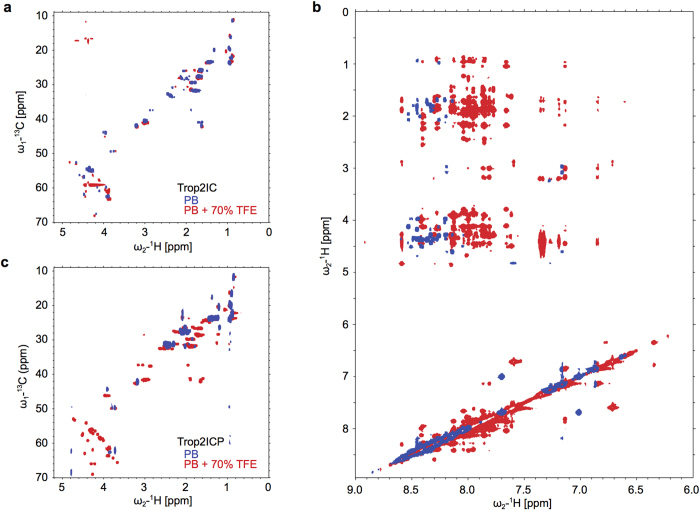
NMR spectra of Trop2IC and Trop2ICP in different solvents. ^13^C-HSQC spectra of peptides in phosphate buffer (PB, blue) and phosphate buffer with 70% TFE (PB + 70% TFE, red) for (**a**) Trop2IC and Trop2ICP (**c**) NOESY spectra overlay of Trop2IC in phosphate buffer (PB, blue) and phosphate buffer with 70% TFE (PB + 70% TFE, red) (**b**).

To compare structural features of Trop2IC and Trop2ICP with respect to secondary structure content, we performed NMR measurements of both peptides in phosphate buffer, pH 7.4, containing 0% or 70% TFE (i.e., no or maximal TFE). Comparison of spectra recorded under both conditions show that the non-phosphorylated form has pre-organized α-helical features. This was confirmed by overlay of spectra in phosphate buffer without and with TFE, where only minor changes were observed (Fig. 3a). However, in phosphate buffer alone, NOE peak intensities are reduced when compared to TFE solution, probably due to fast conformational exchange (Fig. 3b). In the case of the phosphorylated form, we observed reduced solubility leading to poor spectra with pronounced line broadening (Fig. 3c). Therefore, spectra of the phosphorylated form in water and in water/TFE could not be unambiguously compared.

**Figure 4:**
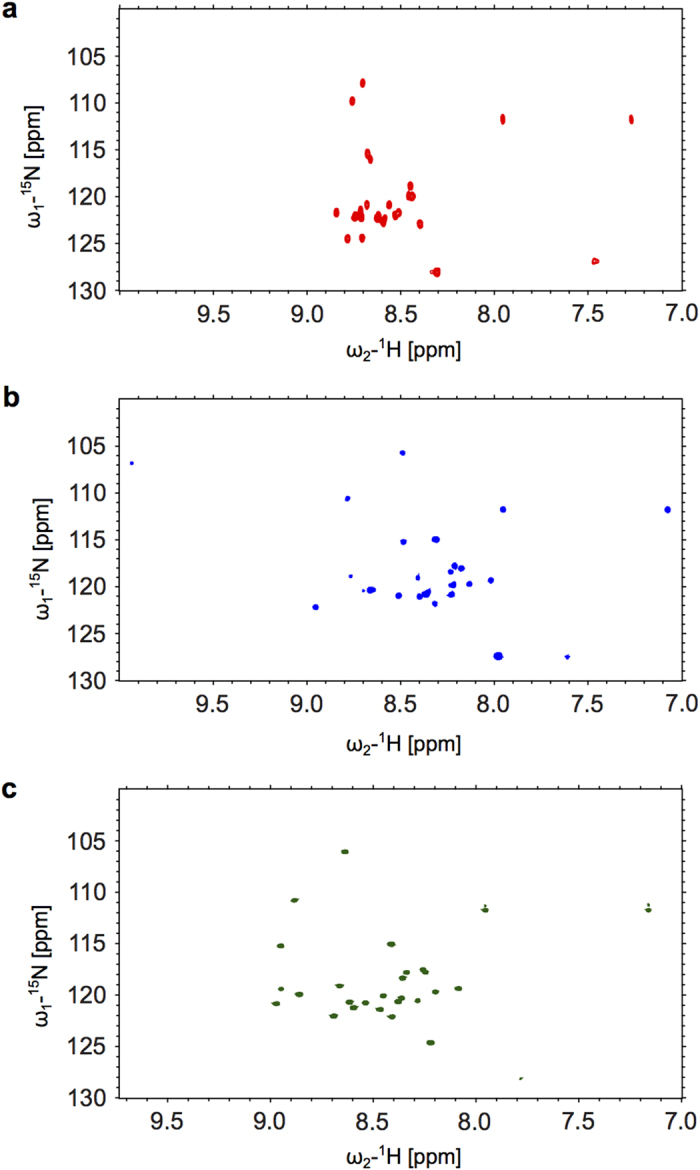
Comparison of ^15^N-HSQC spectra of Trop2IC and Trop2ICP samples in water/TFE solution. (**a**) Trop2IC in 90% H_2_O, 10% D_2_O. (**b**) Trop2IC in 70% TFE, 30% H_2_O. The weak signal at 10 ppm was ascribed to noise. (**c**) Trop2ICP in 70% TFE, 30% H_2_O.

These results were further supported by ^15^N-HSQC NMR spectra of Trop2IC and Trop2ICP under both conditions. Greater signal dispersion was observed for amide groups, indicating better structural organization in a 70% TFE, 30% H_2_O solution (Fig. 4). Chemical shift perturbations upon phosphorylation as calculated from peaks in Fig. 4b and 4c are depicted as Δδ(^1^H,^15^N) per amino acid residue in Supplementary Fig. S1.

### The NMR structure of the cytosolic region of Trop2 reveals a central α-helix

**Figure 5:**
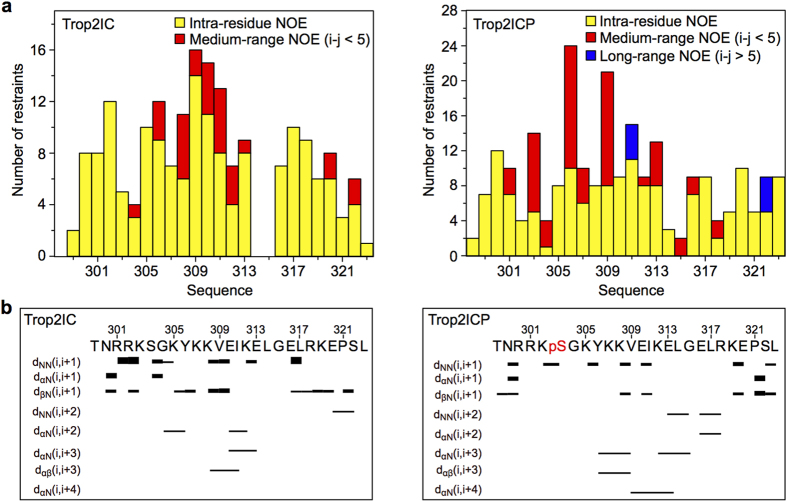
Distribution of NOE restraints per residue and NOE connectivities. (**a**) Distribution of NOE restraints per residue used in structure calculations for both peptides. (**b**) Inter-residue NOE connectivities for both peptides. pS (in red) denotes a phosphoserine residue.

**Figure 6:**
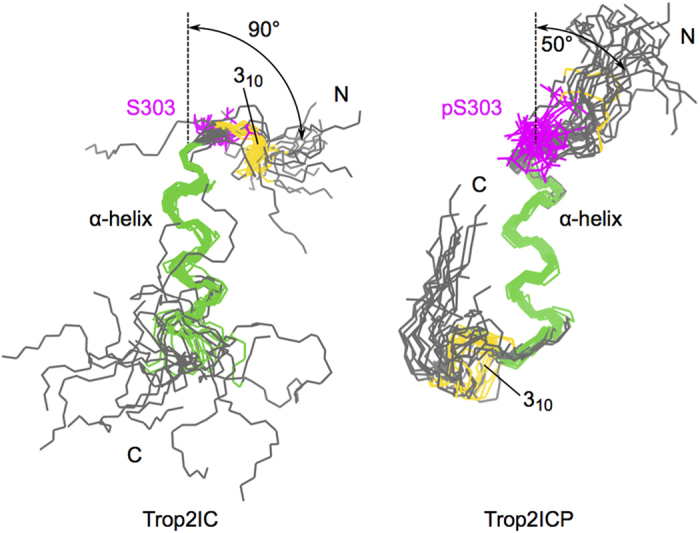
NMR structures of Trop2IC and Trop2ICP. Superposition of twenty lowest energy structures of Trop2IC and Trop2ICP. The α-helical segment is shown in green, segments with a 3_10_ helix conformation are shown in yellow, and the (phospho)Ser303 residue is colored magenta. The central α-helical segment was used for superposition of structures in each ensemble and for orientation of both depicted peptide ensembles.

To define phosphorylation-accompanied structural differences in Trop2IC, we determined the NMR structure of non-phosphoryated and phosphorylated Trop2IC in phosphate buffer pH 7.4 containing 70% TFE. These conditions were found to be favorable for further NMR studies. NOE restraints derived from a series of NOESY spectra together with completeness of resonance assignments (Table 1 and Fig. 5) enabled structure determination of both peptides (Fig. 6).

The central part (Lys305-Leu314) of Trop2IC and Trop2ICP forms an α-helix which is in Trop2IC extended in the C-terminal direction to Arg318, and in Trop2ICP in the N-terminal direction to include the phoshoserine at position 303 (pSer303). Ser303 is solvent exposed in Trop2IC, consistent with observations that serine phosphorylation sites are almost exclusively located in loop and/or flexible regions of protein substrates, enabling kinase access^39^. In the ensemble of Trop2IC structures, an N-terminal 3_10_-helix is present (Arg300-Lys302). In contrast, virtually all structures in the Trop2ICP ensemble exhibit a 3_10_-helix in a different region on the other side of the central α-helix (Glu316-Arg318). However, the most striking differences between Trop2IC and Trop2ICP are the tilt angle relative to the central α-helix, and the degree of order present at peptide termini. While N-terminal regions in Trop2IC and Trop2ICP exhibit similar conformational freedom, their tilt angles are markedly different, corresponding to 90° in Trop2IC and 50° in Trop2ICP. The difference between non-phosphorylated and phosphorylated forms is even greater at their C-termini. In Trop2IC, the C-terminus exhibits significant conformational exchange, while it is significantly more ordered and forms several contacts with the central α-helix in Trop2ICP. The conformation of the Glu320-Pro312 peptide bond was deduced from chemical shifts of Cβ and Cγ^40^. The peptide bond was shown to adopt an exclusively *trans*-conformation, which was additionally confirmed by inspection of corresponding cross-peaks in NOESY spectra.

### Phosphorylation induces structural changes via salt bridge reshuffling

The cytosolic region of Trop2 contains nine basic and four acidic residues, corresponding to half of the polypeptide chain. Since serine phoshorylation introduces an additional negative charge, we expected that charge redistribution could be the main driving force accounting for the observed structural differences between Trop2IC and Trop2ICP.

**Figure 7:**
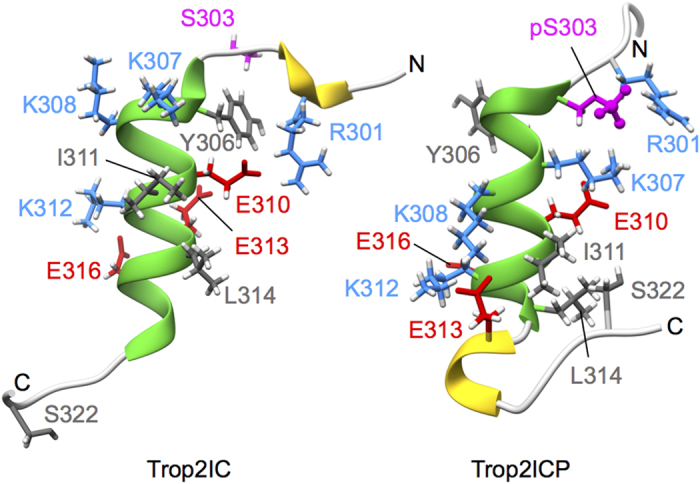
Structures of Trop2IC and Trop2ICP. Side-by-side comparison of representative structures for non-phosphorylated and phosphorylated cytosolic domain of Trop2 reveals salt bridge reshuffling, and formation of a small hydrophobic cluster that underpins the conformational changes of the polypeptide chain. (Phospho)Ser303 is shown in magenta, while acidic and basic residues are shown in red and blue, respectively. Secondary structure elements are color coded as in Fig. 6.

In Trop2IC, the most frequently observed salt bridges in ensemble structures are Glu310-Arg301, which is responsible for the 90° angle between the N-terminal region and the central α-helix, and Lys312-Glu316. Notably, both salt bridges are located on the same side of the C-terminal turn of this helix (Fig. 7). In Trop2ICP, different residue pairs form structure-stabilizing salt bridges. Here, Glu310 forms a salt bridge with Lys307, which is also located within the α-helix but in the N-terminal direction as opposed to the C-terminally located Lys312. The latter forms a salt bridge with neighboring Glu313 within the α-helix, or with Glu316, partially accounting for the upward curvature of the C-terminal region. Also, a salt bridge, Lys308-Glu316, contributes to the observed conformation in the C-terminus of Trop2ICP. Additionally, a hydrophobic cluster involving side chains Ile311, Leu314 and Ser322 is formed, possibly explaining the lower water solubility of the construct upon phosphorylation.

The above described structural differences between Trop2IC and Trop2ICP emanate from the phosphate group attached to Ser303 in Trop2ICP. While in Trop2IC Ser303 is in the vicinity of Tyr306, the side chains of pSer303 and Tyr306 in Trop2ICP point in completely different directions, presumably due to repulsive forces between the negative charge on the phosphate group and the electron-rich ring of Tyr303. Furthermore, while Ser303 is not involved in any significant contacts with other residues in Trop2IC, it forms several ionic contacts in Trop2ICP, where it is predominantly sandwiched between Arg301 and Lys307. Upon phosphorylation of Trop2IC at Ser303, Arg301 is shuffled between a salt bridge with Glu310 and ionic contact with pSer303 (which also involves Lys307). PhosphoSer303 additionally forms a salt bridge with Glu310 in Trop2ICP. In Trop2ICP, Arg301 is not involved in a salt bridge with Glu310, while in Trop2IC this salt bridge governs the above mentioned 90° angle in the N-terminal region.

### Transmembrane helix dimerization could bring the cytoplasmic regions of two Trop2 subunits into close proximity

To analyze the dimerization propensity of the Trop2TM helix, we used molecular dynamics (MD) simulations of two Trop2TM helices embedded in a lipid bilayer. Similar computational approaches have already been reported for studies of membrane-embedded transmembrane (TM) proteins^41^,^42^,^43^,^44^. For example, coarse-grained molecular dynamics (CG MD) simulations of TM segments of glycophorin A and integrins αIIb and β3 have been successfully applied in modeling transmembrane helix oligomers. For glycophorin A, NMR structural analysis and coarse-grained modeling provided a common refined structure of the TM helix homodimer^41^. Recently, an analogous approach has also been used for dimerization studies of TM helices of the Trop2 paralog EpCAM, where it has been demonstrated that TM helices from adjacent subunits of EpCAM dimers preferably form right-handed dimers^7^.

**Figure 8:**
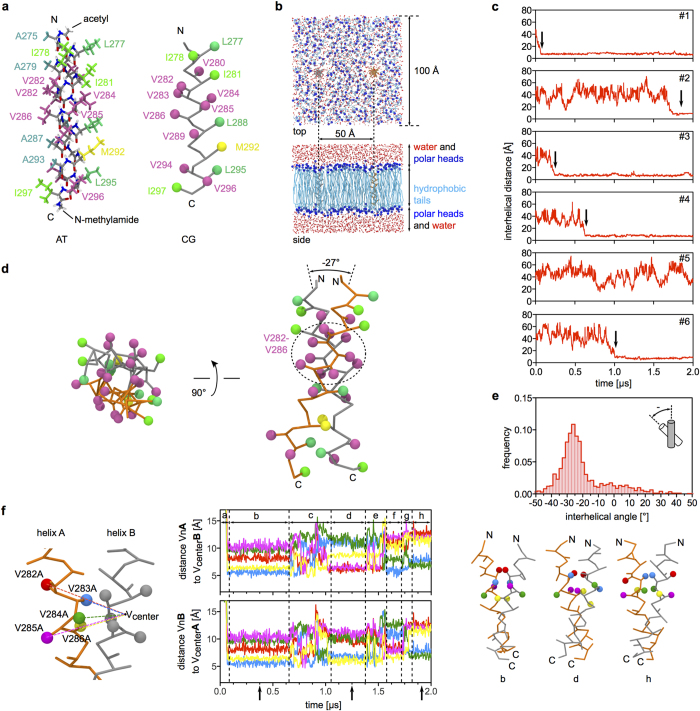
Molecular dynamics simulations of two Trop2 transmembrane helices embedded in a lipid bilayer. (**a**) All-atom (AT) and coarse-grained (CG) model of Trop2TM helix with acetyl and N-methylamide caps on N- and C-termini, respectively. (**b**) Coarse-grained system of two Trop2TM helices (orange, gray) inserted in a lipid bilayer patch composed of 1-palmitoyl-2-oleoylphosphatidylcholine (light and dark blue) surrounded on both sides by water molecules (red). (**c**) Time-course analysis of interhelical distance in six independent 2 μs simulations. Initial close encounters are marked by arrows. (**d**) A central feature of the TM helix dimer is the Val282-Val286 (VVVVV) region directly involved in dimerization as depicted in the representative model. The side chains of amino acid residues of the CG models are shown as spheres colored by residue type. (**e**) Interhelical angle distributions calculated from parts of trajectories in **c** where the two helices form a dimer. (**f**) Time evolution of the VVVVV region in terms of distances of spheres (left) representing individual valine residues within the VVVVV motif in helix A to the center of the juxtaposed motif in helix B and vice versa (center top and bottom, respectively). Three models of the dimer sampled at time points indicated by arrows are shown separately (right).

CG MD simulations of two canonical α-helices, corresponding to the TM segment of Trop2 embedded in a lipid bilayer (Fig. 8a,b) revealed that Trop2TM helices indeed have a propensity to form a transmembrane dimer. The two helices remained close together after an initial encounter (arrows in Fig. 8c) in five of a total of six independent MD simulations (simulations #1-#4 and #6 in Fig. 8c), starting from the same point where the TM helices were separated by 50 Å. The initial encounter is a random event resulting from lateral diffusion of the two TM helices within the lipid bilayer. A representative model of the Trop2TM helix dimer suggests that the central interaction segment is formed by five consecutive valine residues of the ‘VVVVV’ motif (Val282-Val286, Fig. 8d). This motif significantly differs from the GxxxG motif commonly found in TM helix oligomers and other identified motifs^41^,^45^. In EpCAM, a somewhat different motif (VIAVV) forms the dimerization interface^7^. The median relative tilt of TM helical axes observed in calculated trajectories is −27°, corresponding to right-handed packing, and has a unimodal interhelical angle distribution (Fig. 8e). These results are similar to the right-handed packing observed for EpCAM TM dimers, which have a crossing angle of −30° (ref. 7).

To clarify the role of the ‘VVVVV’ motif in Trop2TM helix dimerization, we performed time-evolution analysis of CG MD trajectories. Here, we plotted distances between spheres representing coarse-grained side chains of the individual valine residues of the ‘VVVVV’ motif of one TM helix and the center of the ‘VVVVV’ motif of the juxtaposed helix (and vice versa) of CG MD trajectory #1 vs. time (Fig. 8f, left). This analysis revealed that after initial lateral diffusion (Fig. 8f, center; time range a) the TM helices form a dimer with symmetrical orientation of the juxtaposed ‘VVVVV’ motifs; as revealed by the short distances between Val283 and Val286 and the center of the juxtaposed ‘VVVVV’ motif for both helices. Other symmetrical (time range h) and asymmetrical (time ranges d and f) orientations were observed which are spaced by time ranges where the change in relative rotation of one or both helices around their axis was more frequent (time ranges c, e and g). Similar time plots were obtained for other calculated trajectories. Examples of two symmetrical orientations (from time ranges b and h) and one asymmetrical orientation (from time range d) of the ‘VVVVV’ motifs are shown in Fig. 8f (right). We explain the different orientations by the relatively simple nature of the ‘VVVVV’ motif that makes up more than one turn of the helix. Relative rotation of the helix around its axis results in an equivalent chemical environment at the monomer-monomer interface. The stability of a particular orientation could be affected by interactions between residues of flanking regions, interaction with other (transmembrane) proteins, and lipid bilayer composition. We used 1-palmitoyl-2-oleoylphosphatidylcholine (POPC) to construct our model lipid bilayer; other lipids, with different bilayer thicknesses and area per lipid headgroup could impact TM dimer structure and stability.

Dimerization of Trop2TM could have an important effect on Trop2-mediated signaling, by bringing intracellular regions of laterally interacting Trop2 monomers into close proximity. We observed different relative orientations of TM helices in the dimer with respect to rotation around their axis; however a specific relative orientation could be enforced by the Trop2EC dimer, interaction of Trop2IC with intracellular proteins, interaction of Trop2TM with transmembrane segments of other proteins, binding of PIP2 by Trop2IC, or even by the composition of the lipid bilayer. Since Trop2 is known to be associated with claudin-1 and -7 (ref. 11), and the Trop2 paralogue EpCAM is recruited to tetraspanin-enriched microdomains via interaction with claudin-7 (refs. 14,15), it is quite possible that Trop2 is also recruited to such membrane microdomains. Here, different lipid composition could influence the structure and stability of the Trop2TM dimer.

### The cytoplasmic region of Trop2 may function as a phosphorylation-triggered structural switch

**Figure 9:**
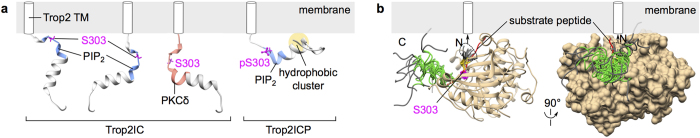
Model of the accessibility of Trop2IC(P). (**a**) Suggested orientation of Trop2IC relative to the membrane with the PIP_2_-binding site (blue) close to the membrane (left) or exposed to the cytosol (middle); in the latter orientation the PKCδ-binding site (salmon) is also easily accessible (right). The hydrophobic cluster formed by structural reorganization of Trop2IC upon phosphorylation suggests a different orientation where the hydrophobic cluster may be in contact with the membrane (far right). The single TM is shown as a cylinder. (**b**) Simulation of the access of PKC to Ser303. For preparation of this model, a structure of the catalytic part of human RAC-alpha serine/threonine kinase^66^ (tan; ribbon representation on the left and surface representation on the right) was used and Ser303 in Trop2IC ensemble models was superimposed on the serine residue of the co-crystallized kinase substrate peptide (red). RAC kinase bears more than 40% identity to the catalytic domains of human PKCα and PKCδ. N and C denote N- and C-termini of Trop2IC, respectively, and the arrow indicates its connection to the TM part. Secondary structure elements of Trop2IC are color coded as in Fig. 6. The singleTM is shown as a cylinder.

To obtain insight into structural changes at the membrane-cytosol interface that accompany Ser303 phosphorylation, it is necessary to consider the Trop2 cytosolic domain in relation to the membrane. From our data, it is not possible to unambiguously define the relative position of the cytosolic part with respect to the TM helix. Similarly, the relative orientation of two IC domains from adjacent Trop2 subunits remains unknown. Therefore, modeling the Trop2TM dimer with two IC regions would be purely speculative. However, since Trop2IC contains a putative PIP_2_-binding site^24^, the orientation of Trop2IC relative to the membrane may be such that this site comes in contact with the membrane (Fig. 9a). In this orientation, access to Ser303 may not be possible. Therefore, access of PKC to Trop2IC would require a different orientation (see Fig. 9a, right), which is similar to the orientation modeled in the inhibitory Trop2IC/PKCδ complex^26^.

The structural changes in Trop2IC associated with Ser303 phosphorylation result in formation of a hydrophobic cluster (Ile311, Leu316, Ser322) which, again, may influence the orientation of Trop2ICP relative to the membrane. This hydrophobic cluster at the C-terminus of Trop2ICP may be in contact with the membrane, however in the proposed orientation (Fig. 9a, far right), the PIP_2_-site would be slightly separated from the membrane. While the described orientations are based solely on proposed Trop2IC functions/interactions, it is tempting to speculate that interactions of Trop2IC with the membrane are influenced by phosphorylation and vice versa, thereby forming an intricate phosphorylation-triggered structural switch at the membrane-cytosol interface. Furthermore, orientation of the cytoplasmic region of Trop2 could be influenced by the dimerization of Trop2TM as discussed above, giving the system a completely new dimension both from the structural point of view as well as from the interaction surfaces with other proteins and membrane components.

Another important aspect of the above described structural features of Trop2IC is its accessibility for phosphorylation by PKCα/β/γ and/or inhibitor-like binding to PKCδ. For visualization purposes we provide a model of PKC access to Trop2IC (Fig. 9b), from which it is clear that access of PKC to Ser303 is indeed possible. However, compared to the (pseudo)substrate-like binding used for modeling inhibitory activity towards PKCδ^26^, in our model Trop2IC only partially occupies the substrate-binding groove of PKC. It is possible that binding to PKC is accompanied by structural changes in the N- and/or C-terminal region near Ser303, resulting in a more extended conformation for Trop2IC, at least in the part of Trop2IC in direct contact with PKC. This is also in line with the previously mentioned instability of the Trop2IC helix in the absence of TFE. In the light of this, Trop2IC could fluctuate between different states depending in the interaction partner. Next, since in (pseudo)substrates and EpCAM, the position equivalent to Trop2 Ser303 is occupied by a basic residue, it is also possible that the negative charge induced by phosphorylation could abrogate the inhibitory activity of Trop2ICP towards PKCδ.

Furthermore, since the cytosolic region of Trop2 forms a signaling complex with β-catenin, it is very likely that interaction between Trop2IC(P) and β-catenin is strongly influenced by the phosphorylation state of the cytosolic part. All of these described structural changes represent an important functional feature and provide a novel insight into regulatory events at the membrane-cytosol interface.

## Methods

### Trop2EC model

A model of the extracellular region of Trop2 (Fig. 1) was prepared using the homology-modeling procedure implemented in MODELLER^46^. As a template, the crystal structure of the extracellular domain of human EpCAM was used (PDB ID 4MZV)^7^. The extracellular regions of Trop2 and EpCAM share 48% sequence identity.

### Sample preparation

Two peptides, a non-phosphorylated and Ser303-phosphorylated variant, corresponding to the Trop2 cytosolic region (T^298^NRRKpSGKYKKVEIKELGELRKEPSL^323^; where pS denotes phosphoserine) were synthesized and purified by GenicBio Limited (China). Both peptides were protected by N-terminal acetylation. Peptide purity (>95%) was analyzed by liquid chromatography and molecular mass was confirmed by mass spectrometry. Peptides were dissolved in Milli-Q water and the molar concentration was determined via absorbance measurements (280 nm) using a molar absorptivity of 1490 M^−1^ cm^−1^.

### Circular dichroism spectroscopy

Circular dichroism (CD) spectra of both peptides (Trop2IC and Trop2ICP) were recorded at 25 °C on an AVIV 60 DS spectropolarimeter with a thermal circulator (±1°C) using a 0.1 cm path-length quartz cell (Hellma, USA). Optical rotation was calibrated using *d*-10-camphorsulfonic acid. Peptides were dissolved to a final concentration of 50 μM in 50 mM sodium phosphate buffer pH 7.40 containing 0–70% (v/v) 2,2,2-trifluoroethanol (TFE; Sigma-Aldrich, USA). All samples were incubated at 4 °C for 12 hours prior to measuring. Averaged spectra were calculated from three separate scans over the range 190–270 nm, and measured with a step size of 0.5 nm and a signal-averaging time of 5 seconds.

CD signals were baseline subtracted (buffer with 0–70% TFE) and converted to mean molar ellipticity per residue [θ] using the following equation (1):
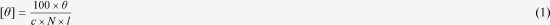
Here, *c* is the peptide concentration (mM), *N* is the number of peptide residues, and *l* is the path-length (cm). Fractional helix content (*f*_*H*_) was estimated using the following equation (2):
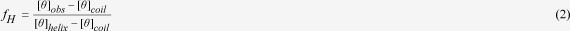
Here, [*θ*]_*obs*_ is the measured mean residue ellipticity at a wavelength of 222 nm, while [*θ*]_coil_ and [*θ*]_helix_ are reference values at the same wavelength for random coil (640 deg cm^2^ dmol^−1^) and α-helical peptides (−42,500 deg cm^2^ dmol^−1^), respectively^47^,^48^.

### NMR spectroscopy

For both peptides, complete sets of homonuclear (NOESY and TOCSY) and heteronuclear (^15^N-HSQC and ^13^C-HSQC) spectra were recorded at 298 K. Samples contained Trop2IC or Trop2ICP peptides at 5 mM concentration in 50 mM sodium phosphate, pH 7.40. For structure calculations, 70% (v/v) TFE was included in the sample buffer. All NMR experiments were recorded on an Agilent-Varian VNMRS 800 MHz NMR spectrometer using an ^1^H/^13^C/^15^N triple resonance cold probe head with inverse detection at 298 K. Chemical shifts were referenced to the internal standard 2,2-dimethyl-2-silapentane-5-sulfonate, DSS (Sigma-Aldrich, USA). Obtained data were processed using the NMRPipe suite^49^ and analyzed using Sparky^50^. Individual amino acid proton spin system assignments were performed using two-dimensional TOCSY experiments (mixing time 80 ms). Sequence-specific assignments were obtained by analysis of NOESY (mixing time 40, 80 and 250 ms) sequential cross peaks connecting neighboring NH and Hα protons. ^13^C and ^15^N chemical shifts were extracted from ^13^C- and ^15^N-HSQC spectra following ^1^H chemical shift assignment from homonuclear NMR experiments.

### Peptide structure calculations and analysis

The standard protocol of CYANA version 3.0 was used for automatic nuclear Overhauser effect (NOE) assignment and structure calculation^51^. CYANA was also used to convert NOE intensities into upper distance restraints according to an inverse sixth power peak volume-to-distance relationship as well as to remove meaningless restraints. The NOE assignment procedure^52^ yielded 139 distance restraints for Trop2IC and 154 for Trop2ICP peptide. For calculation of the Trop2ICP structure, a phosphorylated amino acid library was incorporated^53^. Torsion angle restraints for all residues were derived from chemical shift assignments using TALOS+ software^54^. A high-resolution 3D structure of Trop2IC was determined on the basis of 89 intra-residue, 36 sequential and 14 medium-range distance constraints supported by 30 backbone torsion angle restraints. The high-resolution 3D structure of Trop2ICP was determined using 88 intra-residue, 38 sequential, 24 medium-range and 4 long-range distance constraints, supported by 36 backbone torsion angle restraints (Table 1). The final cycle of CYANA calculations were started with 100 randomized conformers from which 20 conformers with the lowest residual target function values were used for additionally refinement of the results obtained for both peptides. Structure refinement was performed using the explicit solvent model in the YASARA program suite^55^. The refined ensemble structures exhibited good convergence and high definition. Structure validation using PROCHECK-NMR^56^ and WhatIF^57^ demonstrate that the final ensemble of 3D structures is in agreement with distance restraints and displays good geometry and side chain packing. Structure figures were prepared in Chimera^58^. Secondary structure was assigned using STRIDE^59^. Salt bridges were inferred from the orientation of amino acid side chains as calculated from numerous NOE restraints for both phosphorylated and non-phosphorylated peptide variants.

### Molecular dynamics simulations

Molecular dynamics simulations were performed using a coarse-grained (CG) system of two transmembrane helices embedded in a lipid bilayer. First, an all-atom system was constructed where the transmembrane region of Trop2 (A^275^GLIAVIVVVVVALVAGMAVLVI^297^) was modeled as a canonical α-helix. N- and C-termini were capped with acetyl and N-methylamide groups, respectively, to prevent electrostatic repulsion between charged free termini. Two such helices were embedded in a 100 × 100 Å lipid bilayer constructed of 1-palmitoyl-2-oleoylphosphatidylcholine (POPC). Helical axes were oriented perpendicular to the bilayer plane, with an interhelical distance of 50 Å. Next, the system was coarse-grained using a MARTINI force field and solvated^60^. CG molecular dynamics simulations were performed in NAMD^61^ using periodic boundary conditions, a time step of 10 fs and constant temperature (310 K) and pressure (1.01325 bar), using Langevin dynamics. In the initial step, the protein part of the system was fixed and lipids were allowed to equilibrate for 1.25 ns with stepwise harmonic restraint scaling. The equilibrated system was then used as a starting point for six independent 2 μs simulations with the same parameters. Obtained trajectories were analyzed in VMD^62^ and Gromacs^63^,^64^,^65^.

## Author Contributions

M.P., G.I., T.V. and B.L. designed the experiments. M.P., G.I., and T.V. performed the experiments. M.P., G.I., T.V., B.L. and J.P. analyzed the results and wrote the paper.

## Additional Information

**How to cite this article**: Pavšič, M. *et al.* The cytosolic tail of the tumor marker protein Trop2 - a structural switch triggered by phosphorylation. *Sci. Rep.* **5**, 10324; doi: 10.1038/srep10324 (2015).

## Supplementary Material

Supporting Information

## Figures and Tables

**Table 1:** NMR restraints and statistics for the ensemble of 20 lowest energy structures of Trop2IC and Trop2ICP.

	Trop2IC	Trop2ICP
**NOE upper distance limits**^a^		
Intra-residue (|i-j| = 0)	89	88
Sequential (|i-j| = 1)	36	38
Medium-range (|i-j| < 5)	14	24
Long-range (|i-j| > 5)	0	4
Total	139	154
**Torsion angle constrains**		
Backbone (φ/ψ)	30	36
**r.m.s.d. to the mean coordinates (Å)**		
Ordered backbone atoms (298–323)	3.20 ± 1.09	1.81 ± 0.38
Ordered heavy atoms (298–323)	3.94 ± 1.03	2.77 ± 0.38
**Ramachandran plot (125–231)**^b^		
Residues in most favored regions (%)	85.0	84.3
Residues in additional allowed regions (%)	14.0	13.3
Residues in generously allowed regions (%)	0.9	2.3
Residues in disallowed regions (%)	0.0	0.0

^a^None of the 20 structures exhibit distance violations over 0.2 Å and torsion angle violations over 5°.

^b^Ensemble of structures analyzed by PROCHECK-NMR (version 3.4) program^56^.

^c^Ensemble of structures validated and analyzed using WhatIF^57^.
