# Supplementary material for: The cytosolic tail of the tumor marker protein Trop2 - a structural switch triggered by phosphorylation
Source: Sci Rep. 2015 May 18;5:10324. doi: 10.1038/srep10324 (PMC4434849; doi:10.1038/srep10324)
Supplement: Supporting Information [file srep10324-s1.pdf]

Supplementary Information

The cytosolic tail of the tumor marker protein Trop2 - a structural switch triggered by phosphorylation

Miha Pavšič, Gregor Ilc, Tilen Vidmar, Janez Plavec & Brigita Lenarčič

Correspondence should be addressed to B.L. (brigita.lenarcic@fkkt.uni-lj.si)
and M.P. (miha.pavsic@fkkt.uni-lj.si).

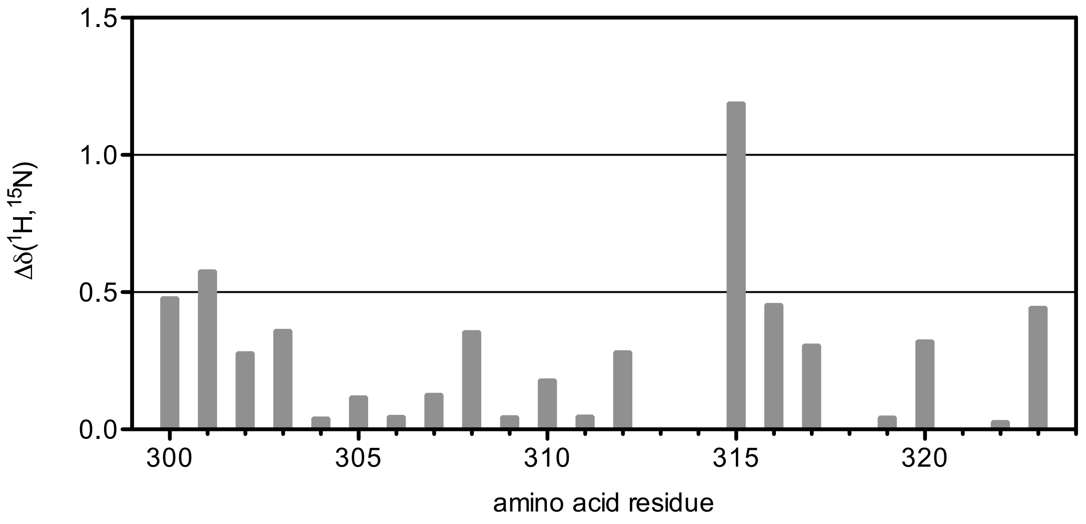

Supplementary Figure S1 $\Delta\delta(^1H,^{15}N)$ values for non-phoshorylated (Trop2IC) and phosphorylated (Trop2ICP) peptide variants (in 70% TFE, 30% H_2O) calculated using the following formula: $\Delta\delta(^1H,^{15}N) = ((^1H_{Trop2IC}-^1H_{Trop2ICP})^2 + (0.154*(^{15}N_{Trop2IC}-^{15}N_{Trop2ICP}))^2)^{0.5}$.
